# Efficacy analysis and adverse event management of selinexor in the treatment of relapsed/refractory multiple myeloma

**DOI:** 10.3389/fmed.2026.1927209

**Published:** 2026-10-06

**Authors:** Zengli Kuang, Yu Chen, Cuiai Ren

**Affiliations:** Weifang People's Hospital, Shandong Second Medical University, Not applicable Weifang, Shandong, China

**Keywords:** adverse reaction management, clinical application, efficacy analysis, relapsed/refractory multiple myeloma, selinexor

## Abstract

The treatment of relapsed/refractory multiple myeloma (RRMM) remains a significant clinical challenge, particularly for patients with multidrug resistance, who have a poor prognosis and limited therapeutic options. Selinexor, as the world's first oral selective inhibitor of nuclear export (SINE), targets the XPO1 protein, offering a novel treatment mechanism distinct from traditional agents for RRMM. This narrative review systematically synthesizes the mechanism of action of Selinexor and critically evaluates evidence from key clinical studies (such as the STORM and BOSTON trials). It focuses on discussing its clinical positioning across different scenarios and its combination strategies with commonly used regimens (e.g., SVd). It also addresses standardized, proactive management pathways for its characteristic adverse effects (e.g., thrombocytopenia, gastrointestinal reactions). These strategies are tailored to patient stratification (such as triple-/penta-refractory, elderly/frail patients). The aim is to provide clinicians with a practical, evidence-based reference for the safe and effective application of this drug to optimize treatment outcomes for RRMM patients.

## 1 Introduction

Multiple myeloma (MM) is the second most common hematologic malignancy, accounting for approximately 10% of all hematologic cancers, and is characterized by the malignant proliferation of clonal plasma cells in the bone marrow (1–3). The incidence of this disease increases with age, with a median age at diagnosis of approximately 70 years. As the global population ages, the disease burden of MM is increasing. Epidemiological data indicate that the age-standardized incidence of MM in China is approximately 1–2 per 100,000, which is lower than in Western countries. However, this rate has shown a significant upward trend in recent years, placing a heavy burden on patients’ families and the healthcare system (4, 5).

Over the past two decades, landmark progress has been made in MM treatment. The emergence of novel agents, such as proteasome inhibitors (PIs, e.g., bortezomib, carfilzomib), immunomodulatory drugs (IMiDs, e.g., lenalidomide, pomalidomide), and monoclonal antibodies (mAbs, e.g., anti-CD38 daratumumab), has revolutionized treatment outcomes (6). The median overall survival (OS) for newly diagnosed patients has significantly extended from the traditional 3–5 years to 8–10 years (7). However, MM is still considered an incurable disease, with a disease course often characterized by cycles of “remission-relapse,” making the relapsed/refractory stage a major clinical challenge (8). Consequently, the research and development of new drugs with novel mechanisms of action for RRMM are crucial to overcome the increasingly severe challenge of multidrug resistance (9–11).

XPO1 (Exportin-1), also known as Chromosome Region Maintenance 1 (CRM1), is a member of the karyopherin-*β* family of nuclear transport proteins and the primary receptor that mediates protein export from the nucleus. It is responsible for the nuclear export of more than 220 cargo proteins, including multiple types of tumor suppressor proteins (12). XPO1 is significantly overexpressed in a variety of hematologic malignancies, including multiple myeloma (MM). Its aberrant activation drives tumor progression via a dual mechanism—tumor suppressor inactivation coupled with oncogene activation—which makes XPO1 an ideal therapeutic target for overcoming multidrug resistance (12, 13).

While the clinical application of Selinexor is currently approved for RRMM, it is encouraging to note that its potential is not limited to this indication. As the first-in-class SINE, Selinexor is actively being investigated across a broad range of both hematologic malignancies and solid tumors, highlighting its larger therapeutic potential as a novel anti-cancer agent (14).

## 2 A new paradigm for RRMM treatment based on XPO1 inhibition

### 2.1 The challenge of RRMM drug resistance and the urgency of new drug development

According to the International Myeloma Working Group criteria, relapsed MM refers to disease progression after a period of remission, while refractory MM is defined as no response to current therapy or disease progression within 60 days during or after treatment (15). Multidrug resistance, where patients become refractory to drugs with major mechanisms of action, is a particularly challenging issue. Among these, triple-class refractory (refractory to at least one PI, one IMiD, and one anti-CD38 mAb) and penta-refractory (refractory to at least two PIs, two IMiDs, and one anti-CD38 mAb) patients have a very poor prognosis, with OS sharply decreasing to approximately one year or even less (15, 16).

The mechanisms of resistance are complex, mainly including: (1) clonal evolution and heterogeneity of tumor cells, generating resistant subclones under treatment pressure; (2) protection by the bone marrow microenvironment, which provides a survival niche for myeloma cells; (3) compensatory activation of alternative signaling pathways (e.g., NF-*κ*B, PI3 K/AKT); and (4) alterations in drug targets, such as upregulation of immunoproteasome subunits or changes in cereblon expression (17–20). To address these resistance mechanisms, developing drugs with novel mechanisms of action is crucial to overcoming treatment bottlenecks (21, 22).

### 2.2 Theoretical basis for exportin XPO1 as a new target

XPO1 (Exportin-1/CRM1) is a member of the karyopherin-*β* family of nuclear transport proteins and is the primary receptor for protein export from the nucleus (12). Under physiological conditions, XPO1 recognizes and binds cargo proteins containing a classic leucine-rich nuclear export signal (NES). Driven by energy from the RanGTPase cycle, it transports proteins from the nucleus to the cytoplasm, a process essential for normal cellular functions including cell cycle regulation, stress response, and gene expression.

However, XPO1 is significantly overexpressed in various hematologic malignancies and solid tumors, including multiple myeloma (13). This overexpression drives tumor progression primarily through two mechanisms: firstly, inactivation of tumor suppressor proteins (e.g., p53, p73, FOXO) by excessive nuclear export; secondly, enhanced translation of oncoprotein mRNAs (e.g., c-Myc, Bcl-2, Bcl-6). This imbalance of “tumor suppressor inactivation + oncogene activation” makes XPO1 a promising target for overcoming drug resistance. Theoretical studies suggest that inhibiting XPO1 can simultaneously restore multiple tumor suppressor pathways and block oncogenic signals, offering a new strategy for multidrug-resistant patients (23–26).

### 2.3 Mechanism of action of selinexor

Selinexor, a first-in-class oral Selective Inhibitor of Nuclear Export (SINE), exerts its anti-tumor effects through covalent binding to the Cys528 residue within the substrate-binding groove of XPO1. This binding occurs via the triazole ring structure of selinexor, forming an irreversible covalent bond that permanently blocks the nuclear-export-signal (NES)-dependent nuclear export function of XPO1 (27, 28). This irreversible binding mechanism distinguishes selinexor from reversible XPO1 inhibitors, ensuring sustained target occupancy and prolonged pharmacodynamic effects. This core mechanism fundamentally reverses the abnormal nucleocytoplasmic transport balance in myeloma tumor cells and triggers multi-pathway anti-tumor responses (28). Primarily, XPO1 inhibition leads to three major downstream biological consequences.

(1) Nuclear retention and reactivation of Tumor Suppressor Proteins (TSPs): When XPO1 function is inhibited, TSPs such as p53, p21, and p27, as well as I*κ*B (an inhibitor of NF-*κ*B signaling), accumulate in the nucleus. Elevated nuclear p53 levels transcriptionally activate apoptotic pathways; accumulation of I*κ*B inhibits the pro-survival NF-*κ*B signaling pathway. These actions synergistically induce G1 cell cycle arrest and caspase-dependent apoptosis in tumor cells (27).
(2) Nuclear retention and reduced translation of oncoprotein mRNAs: mRNAs of oncogenes like c-Myc and Bcl-2 are retained in the nucleus, preventing their translation in the cytoplasm, leading to decreased levels of these key pro-cancer proteins and further weakening tumor cell proliferation and anti-apoptotic capacity (24, 27).
(3) Inhibition of DNA damage repair: Selinexor can also interfere with the nucleocytoplasmic transport of DNA damage repair-related proteins, disrupting the repair process, increasing genomic instability, and making tumor cells more sensitive to apoptotic signals (29).

Importantly, the anti-tumor activity of selinexor is not exclusively dependent on functional wild-type p53. In drug-resistant myeloma carrying TP53 deletion or mutation [del(17p)], conventional proteasome inhibitors and IMiDs largely rely on intact wild-type p53 signaling to trigger tumor cell apoptosis and thus show poor clinical efficacy. Selinexor, however, promotes nuclear retention of the related tumor suppressor family protein p73 even in cells with complete TP53 loss. Mechanistically, nuclear p73, a structural and functional homolog of p53, binds to p53 consensus DNA-binding sites and transcriptionally activates pro-apoptotic genes such as PUMA and NOXA, which initiates G1-phase cell-cycle arrest and caspase-dependent apoptosis. FOXO3a also plays a critical role in this process, mediating selinexor-induced PUMA expression. This provides a mechanistic explanation for the meaningful clinical benefit observed in del(17p) high-risk RRMM patients treated with selinexor-containing regimens (28, 30, 31).

Beyond the TP53-family signaling pathway, selinexor counteracts other high-risk cytogenetic drivers. In myeloma with 1q21 amplification, elevated XPO1 expression continuously exports multiple tumor suppressors out of the nucleus to sustain oncogenic proliferation; selinexor-driven nuclear retention reverses this phenotype. For t(4;14) translocation-positive high-risk myeloma, XPO1-dependent cytoplasmic export inactivates FOXO3a; selinexor restores nuclear FOXO3a accumulation and suppresses tumor cell proliferation (28). These mechanistic insights collectively support the clinical observation that selinexor-based regimens provide clinically meaningful PFS benefits for patients with high-risk cytogenetic abnormalities, as demonstrated in subgroup analyses of the BOSTON study (31).

More recent research has revealed additional layers of complexity. Selinexor can modulate non-apoptotic cell death pathways in conjunction with autophagy (32) and exert immunomodulatory effects by enhancing CD8+ T and NK cell activity while remodeling the tumor microenvironment. Specifically, selinexor has been shown to enhance CD8+ T lymphocyte and NK cell activation, repolarize macrophage phenotypes, and suppress myeloid-derived suppressor cells (MDSCs). In bone marrow samples from treated patients, increased expression of CD8 and granzyme B in T cells has been observed following selinexor treatment (33, 34). Furthermore, selinexor can relieve the inhibitory state of NK cells, enhancing their anti-tumor activity while reducing the production of pro-inflammatory cytokines. These immunomodulatory effects provide a theoretical foundation for combining selinexor with immune checkpoint inhibitors, CAR-T cells, and bispecific antibodies (33–35). Furthermore, selinexor has demonstrated potential as a bridging strategy prior to CAR-T therapy, with mechanistic rationale detailed in Section 2.4 (34). Furthermore, from the perspective of the tumor hypoxic microenvironment, Selinexor has demonstrated the ability to synergize with other agents like venetoclax to overcome bortezomib resistance, suggesting its potential to modulate the microenvironment and reverse drug resistance (36) (Figure 1).

**Figure 1:**
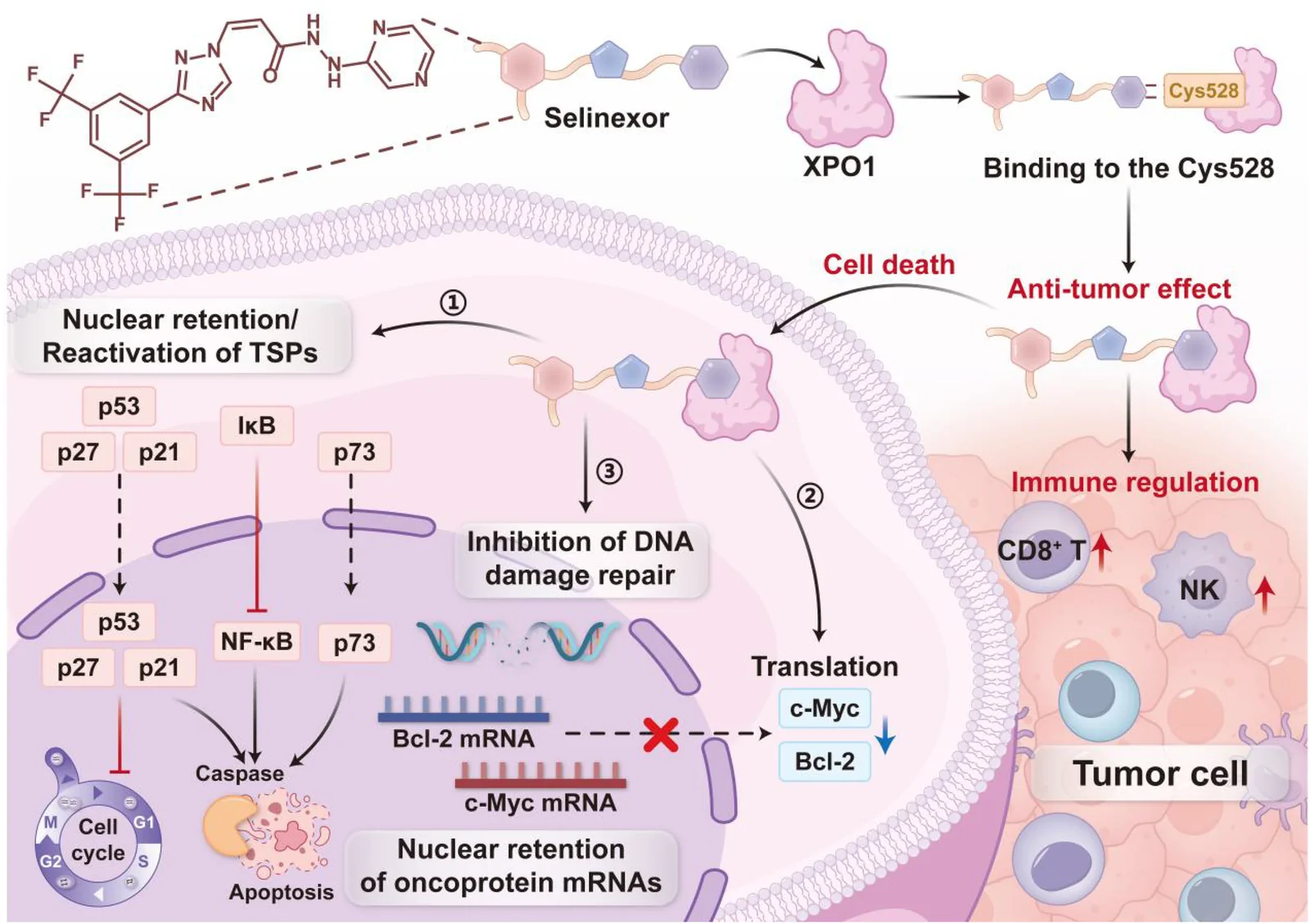
Schematic diagram of selinexor's anti-tumor mechanism through XPO1 inhibition.selinexor covalently binds to the Cys528 site of XPO1 (28), blocking nuclear export. This triggers three main events: (1) Tumor suppressor proteins (p53, p21, p27, and I*κ*B) accumulate in the nucleus, inhibiting the NF-*κ*B signaling pathway, and together they induce apoptosis and G1 phase arrest. Even in the absence of TP53, it can still induce apoptosis by activating p73 (28, 30, 31); (2) Oncogene c-Myc and Bcl-2 mRNAs are retained in the nucleus, reducing their translation (24, 27); (3) DNA repair is disrupted by interfering with the transport of repair proteins (29). Additionally, selinexor can reshape the tumor microenvironment by enhancing CD8⁺ T cell and NK cell activity (33, 34).

Nevertheless, preclinical data also highlight potential resistance mechanisms: acquired mutations at the XPO1-Cys528 binding site can abolish covalent drug attachment and produce primary selinexor resistance (28). Additionally, as discussed in Section 2.4, the RNA-binding protein hnRNPU has been identified as a potential biomarker for selinexor sensitivity, where high hnRNPU expression correlates with reduced treatment efficacy (37). These findings underscore the importance of ongoing biomarker-driven research to optimize patient selection and overcome resistance.

### 2.4 Synergistic mechanisms with standard-of-care drugs and resistance pathways

Selinexor shows rational, mechanism-based synergy with standard MM therapies. It enhances dexamethasone efficacy by blocking NF-*κ*B and increasing nuclear retention of the glucocorticoid receptor (38). It works synergistically with PIs by eliminating the compensatory activation of the NF-*κ*B pathway that often occurs in response to proteasome inhibition, providing the mechanistic basis for the successful BOSTON study (SVd regimen) (39). It induces apoptosis independently of the IMiD target cereblon, offering a strategy for IMiD-resistant patients (30).

However, primary or acquired resistance can emerge through multiple mechanisms. A recent basic research study revealed a potential mechanism: the RNA-binding protein hnRNPU, whose expression correlates with resistance, weakens the efficacy of selinexor by affecting the localization of XPO1 cargoes and the translation of key proteins. Patients sensitive to selinexor had lower hnRNPU expression, suggesting that hnRNPU may serve as a potential biomarker for predicting selinexor efficacy, providing a new direction for personalized treatment in the future (37).

Beyond hnRNPU, additional biomarkers have been identified as predictors of selinexor response. Somatic XPO1 Cys528 point mutations can abolish covalent drug binding, leading to primary resistance (28). Phosphorylated STAT3 (p-STAT3) has been shown to predict selinexor resistance, with combination of selinexor and a STAT3 inhibitor representing a potential strategy for resistant cases (40). The TGF*β*-SMAD4 pathway has also been identified as a mediator of selinexor resistance, with higher pathway activity correlating with prolonged progression-free survival (41). Furthermore, selinexor upregulates BCMA expression on myeloma cells in a time- and dose-dependent manner, with 50 nM selinexor treatment for 5 days increasing BCMA receptor density by 4.2-fold in OPM-2 cells, potentially augmenting BCMA-directed CAR-T cell function, providing a mechanistic rationale for its use as a bridging regimen prior to CAR-T therapy (34, 42). This multi-biomarker framework complements single-indicator evaluations and provides a basis for individualized patient selection in clinical practice.

## 3 Analysis of clinical efficacy evidence for selinexor in treating RRMM

### 3.1 Pivotal clinical studies and key insights

A series of pivotal clinical studies have established the role of selinexor in the treatment of RRMM; key efficacy data are summarised in Table 1. The STORM study (30), a single-arm phase II trial, enrolled 122 patients with triple-class refractory (refractory to PI, IMiD and anti-CD38 mAb) RRMM and treated them with the Xd regimen (selinexor plus dexamethasone). The overall response rate (ORR) was 26.2%, and median progression-free survival (PFS) was 3.7 months. This study provided the first proof-of-concept that XPO1 inhibition can induce clinically meaningful responses even in a heavily pretreated, multidrug-resistant population, thus establishing the evidence base for selinexor in the late-line setting. However, it is important to note that STORM was a single-arm, open-label Phase II study lacking a randomized control group. The enrolled patients were heavily pretreated (median 7 prior lines of therapy) with triple-class refractory disease, which substantially limits the generalizability of its findings. Furthermore, the initially used selinexor 80 mg twice-weekly dosing regimen was associated with a high incidence of grade ≥3 adverse events (thrombocytopenia 60%), which subsequently prompted the exploration of optimized dosing schedules (30, 43).

**Table 1:** Summary of efficacy data from key clinical studies of selinexor-based regimens in RRMM.

Study Name	Regimen	N (Exp/Cont)	ORR (%)	Median PFS (months)	Key Notes
STORM (30)	Xd	122	26.2	3.7	Single-arm; triple-class refractory
BOSTON (31)	SVd/Vd	195/207	76.4/62.3 (*p* < 0.001)	13.93/9.46 (HR 0.70)	Pivotal Phase 3; lenalidomide-refractory
GEM-SELI BORDARA (44)	S-DVd	57 (24 Part1;33 Part2)	Part1 overall:50; Part2 overall:82	Part1 overall:7.2; Part2 overall:25.1	Non-randomized Phase 2; Part1 (≥3 prior lines); Part 2 (≥1 prior line)
STOMP (45) (XKd)	XKd	32	78	12.5	Phase 1b/2; heavily pretreated (median 4 prior lines)

Exp/Cont, experimental/control; PFS, progression-free survival; HR, hazard ratio; ORR, overall response rate; PI, proteasome inhibitor.

Subsequently, the phase III randomised BOSTON study (31) enrolled 402 lenalidomide-refractory RRMM patients and compared the SVd regimen (selinexor + bortezomib + dexamethasone) with standard Vd. The SVd arm showed significantly prolonged median PFS (13.93 vs. 9.46 months; HR = 0.70, *p* < 0.001) and higher ORR (76.4% vs. 62.3%, *p* < 0.001). This trial served as the pivotal registration study and supports the use of selinexor in earlier relapses. Nevertheless, several methodological limitations should be acknowledged. The BOSTON trial was an open-label, non-blinded study, which may introduce subjective bias in response evaluation and adverse event recording. Moreover, the trial excluded patients with severe organ dysfunction, active infections, and extremely frail elderly patients, resulting in a highly selected study population that may not fully represent unselected real-world RRMM cohorts. Additionally, no prospective head-to-head trials have compared the SVd regimen with other standard-of-care regimens such as DVd (daratumumab + bortezomib + dexamethasone) or Kd (carfilzomib + dexamethasone), and indirect cross-trial comparisons are confounded by differences in baseline patient characteristics and prior treatment lines (31, 43). Notably, the BOSTON regimen has since been optimised for better tolerability (see Section 3.2).

More recent trials have explored combining selinexor with monoclonal antibodies. The GEM-SELIBORDARA study (44), a non-randomised phase II trial, evaluated S-DVd (selinexor + daratumumab + bortezomib + dexamethasone) in RRMM patients. A total of 57 patients were enrolled in two parts: Part 1 (*n* = 24, heavily pretreated with ≥3 prior lines) and Part 2 (*n* = 33, early relapse with ≥1 prior line). In Part 1, the ORR was 50% with a median PFS of 7.2 months; 17 of 24 patients (71%) were double-class refractory, and nearly all patients (23/24) were lenalidomide-refractory. In Part 2, the ORR was 82% with a median PFS of 25.1 months; post-hoc subgroup analysis within Part 2 showed a median PFS of 22.8 months among lenalidomide-refractory patients in this early-relapse cohort. Notably, while the STOMP study (45) explored XKd (selinexor, weekly carfilzomib, dexamethasone) in 32 heavily pretreated patients (median 4 prior lines), showing an ORR of 78% and median PFS of 12.5 months; however, its small sample size (*N* = 32) limits definitive conclusions. Compared with other standards like DVd or Kd, no head-to-head trials exist with SVd; however, the unique all-oral, once-weekly schedule of the optimized SVd regimen offers a distinct convenience advantage over regimens requiring multiple infusions.

A 2024 meta-analysis of 11 studies including 1087 RRMM patients (46) further quantified the overall efficacy: clinical benefit rate 56.21% (95% CI: 44.58%–67.29%) and complete response rate 4.89% (95% CI: 2.41%–9.71%).

Notably, all pivotal selinexor trials possess inherent methodological limitations that restrict the generalizability of clinical evidence. The STORM and BOSTON trials are open-label, non-blinded studies, which introduces subjective bias in response evaluation and adverse event recording. Furthermore, most registered trials excluded patients with severe organ dysfunction, active infection, and extremely frail elderly patients, resulting in a highly selected study population that cannot fully represent unselected real-world RRMM cohorts. In addition, no prospective head-to-head trials have compared selinexor-based regimens with novel immunotherapies (bispecific antibodies, CAR-T cells), and indirect cross-trial comparisons are confounded by differences in baseline patient risk stratification and prior treatment lines, leading to unresolved uncertainty regarding its optimal clinical positioning in the modern RRMM treatment landscape.

### 3.2 Optimisation pathways: timing, combination and dosing

#### 3.2.1 Strategic advantages of “early intervention” and “combination therapy”

A systematic review and meta-analysis by Gu et al. (47), including 16 studies with 817 patients, clarified the significant impact of treatment timing and combination strategy on efficacy. The results showed that using selinexor earlier (before the fifth line of therapy) resulted in a significantly higher ORR [odds ratio (OR) 2.15, 95% CI: 1.38–3.35] and longer PFS (HR 0.67, 95% CI: 0.51–0.88) compared to later-line salvage therapy. Concurrently, triple-drug regimens combining selinexor with a PI or IMiD showed significantly superior efficacy compared to doublet regimens (OR 2.43, 95% CI: 1.56–3.78). These findings confirm the clinical advantages of early intervention and combination therapy strategies (47).

#### 3.2.2 Optimization of dosing regimens

The initially approved twice-weekly (BIW) dosing regimen of selinexor (60 mg BIW), while effective, was associated with a high incidence of adverse events such as thrombocytopenia, nausea, and fatigue, limiting its widespread use. A 2025 review by Baljevic et al. (48) systematically summarized evidence for dosing regimen optimization: using selinexor at 100 mg once weekly (QW) in the SVd regimen maintained efficacy while significantly improving tolerability; for elderly or frail patients, starting doses could be reduced to 60 mg/week or even 40 mg/week while maintaining treatment benefit. These optimised regimens reduced treatment discontinuation due to adverse events from 25%–30% to 10%–15% (48).

Real-world data from the Greek cohort by Kastritis et al. (49) validated this approach, showing that patients receiving the QW regimen had significantly lower rates of grade 3–4 thrombocytopenia (28% vs. 52%) and fewer discontinuations due to adverse events (12% vs. 28%) than those receiving the BIW regimen, with no significant difference in ORR (62% vs. 58%). This real-world validation confirms that the optimized QW dosing strategy is both effective and better tolerated outside the controlled environment of clinical trials.

### 3.3 Real-world evidence and stratified management for special populations

Prospective clinical trials enroll highly selected patients, which may not fully reflect drug performance in heterogeneous real-world populations. Multiple real-world retrospective cohorts from Europe, the United States and China have addressed this gap by providing complementary insights into unselected routine-practice populations.

#### 3.3.1 Elderly/frail patients

Elderly patients (≥75 years) with poor physical tolerance are underrepresented in prospective trials. Real-world cohort data demonstrate that individualised low-dose once-weekly selinexor regimens achieve an ORR of 55%–60% in this population, comparable to that observed in younger patients, while the incidence of grade 3–4 AEs is controlled below 35%. Baljevic et al. (48) further reported that, with proactive dose adjustment (e.g., starting at 40 mg/week for very frail individuals) and enhanced supportive care, the incidence of grade 3–4 AEs can be reduced from 58% to 32% and discontinuation due to AEs from 22% to 9%. These findings confirm that optimised dosing significantly improves treatment persistence in elderly/frail subgroups without compromising efficacy. Nevertheless, real-world evidence for profoundly frail multi-comorbid patients remains limited; most available retrospective cohorts exclude patients with very poor performance status.

#### 3.3.2 Renal impairment

For RRMM patients with renal impairment (eGFR <60 mL/min/1.73m^2^), selinexor offers a distinct advantage, as its clearance is primarily hepatic and exhibits no renal-dependent metabolism. Real-world data confirm that adjusted-dose selinexor-based regimens sustain effective disease control without increased renal toxicity or cumulative systemic adverse events, supporting its safe application in this high-risk comorbid population that is rarely systematically evaluated in prospective trials. Preliminary real-world data from the Karyopharm Expanded Access Program (KEAP) have evaluated selinexor-based regimens in patients with RRMM and dialysis-dependent renal impairment, reporting encouraging safety and efficacy signals (50). However, these data originate from a small-sample retrospective cohort and have only been presented in abstract form; dedicated prospective studies in this population remain lacking (50).

#### 3.3.3 High-risk cytogenetics

Patients with high-risk cytogenetic abnormalities such as del(17p), t(4;14), t(14;16), or 1q amplification, including the recently defined concepts of “double-hit” and “triple-hit” myeloma (harboring two or three of these abnormalities) (51), typically respond poorly to conventional therapies. The BOSTON study analysis demonstrated that the SVd regimen provided clinically meaningful PFS benefits for patients with high-risk cytogenetic abnormalities compared to the Vd regimen. The underlying mechanism is discussed in detail in Section 2.3 – notably, selinexor can promote nuclear retention of p73 to bypass TP53-deficiency-related apoptosis resistance, which underpins the observed clinical activity (28, 30, 31). While BOSTON subgroup analyses demonstrate PFS benefit for high-risk cytogenetic patients receiving SVd versus Vd, subgroup analyses are exploratory by statistical design and cannot substitute for dedicated prospective validation in pure high-risk myeloma cohorts.

A retrospective single-center study (52) evaluated 60 RRMM patients treated with selinexor-based regimens, including 40 with high-risk cytogenetics (16 with del17p and 24 with other high-risk abnormalities) and 20 with standard-risk cytogenetics. The objective response rate was 50% in the del17p group, 41.7% in the other high-risk group, and 35% in the standard-risk group (*p* = 0.71). Median overall survival was comparable across groups: 10.9 months for del17p, 10.3 months for other high-risk, and 10.3 months for standard-risk (*p* = 0.92). Notably, patients receiving selinexor as bridging therapy prior to subsequent treatment had a median OS of 15.5 months versus 9 months for those receiving selinexor for other reasons. These findings suggest that selinexor-based regimens yield similar outcomes across cytogenetic risk groups, providing real-world validation of the p73-mediated bypass mechanism discussed in Section 2.3. It should be noted that sample sizes for double-hit/triple-hit subgroups are small, and conclusions require further large-cohort confirmation.

#### 3.3.4 Extramedullary disease (EMD)

Selinexor holds unique advantages in treating multiple myeloma with extramedullary disease. Its mechanism of action directly targets tumor cells without relying on the bone marrow microenvironment, effectively overcoming the characteristics of EMD cells, which are highly aggressive and less dependent on the bone marrow niche (53). Clinical data from Yin et al. (54) using the XVRd regimen (selinexor combined with bortezomib, lenalidomide, and dexamethasone) in newly diagnosed high-risk multiple myeloma patients with EMD showed a 100% ORR, with 80% achieving deep response (≥very good partial response). The median time to first response for extramedullary lesions was only 1.2 months. These findings suggest that for RRMM patients presenting with extramedullary involvement, a selinexor-containing regimen should be considered as a preferred salvage option (53, 54). Of note, encouraging EMD-related efficacy signals originate mostly from small-sample single-arm cohorts; dedicated randomized trials focusing on EMD-RRMM are absent, and responses are not universally durable across all EMD patients.

#### 3.3.5 Triple-/penta-refractory and lenalidomide-refractory patients

For patients resistant to all major drug classes (PI, IMiD, anti-CD38 mAb), treatment options are extremely limited. Data from STORM (30) and other studies support the Xd regimen as an effective salvage option, providing new therapeutic opportunities for patients with limited remaining choices. For lenalidomide-refractory patients, the BOSTON study (31) established the SVd regimen as a standard treatment option. The choice between SVd and other regimens (e.g., DVd, Kd) should be individualized based on prior therapies, resistance profile, convenience (QW oral vs. intravenous), and toxicity. The GEM-SELIBORDARA study (44) enrolled 57 patients in two parts, in the double-class refractory subgroup (concentrated in Part 1), selinexor-based regimens still demonstrated meaningful activity, supporting the mechanistic rationale that XPO1 inhibition bypasses cross-resistance to PIs and IMiDs through distinct pathways. Notably, real-world OS data for penta-refractory subgroups remain sparse, and STORM exclusively represents heavily pre-treated populations; how Xd performs in earlier-line triple-class refractory settings remains undetermined.

#### 3.3.6 Proactive adverse-event management and impact on outcomes

Beyond efficacy, real-world evidence strongly supports the value of proactive adverse event (AE) management. A best practices (BP) program implemented at Florida Cancer Specialists & Research Institute (55)—featuring selinexor initiation at doses ≤80 mg once weekly and upfront use of standardized antiemetics—significantly improved treatment outcomes. In the 12-month post-BP period, 41 patients received selinexor-based therapy compared to 68 patients in the pre-BP period. Patients treated in the post-BP period had a significantly reduced risk of treatment failure (HR = 0.50; 95% CI: 0.27–0.92), and those in the pre-BP period were four times more likely to discontinue therapy (OR = 4.0; 95% CI: 1.75–9.3). These findings indicate that a structured BP program tailored to selinexor can increase time to treatment failure, extend treatment duration, and lower the incidence of dose-limiting toxicities.

#### 3.3.7 Chinese multicentre real-world experience

A Chinese multicenter real-world study (56) involving 53 RRMM patients treated with selinexor-based regimens across 11 centers reported an ORR of 44.7% (21/47 evaluable patients), including 6.4% complete remission, 8.5% very good partial remission, and 29.8% partial remission. The median number of prior lines of therapy was 4 (range: 1–11), with 64.2% of patients being double-class refractory and 37.7% triple-class refractory. Among evaluable patients, 66.7% had high-risk cytogenetics at diagnosis. This study provides important real-world evidence for East Asian RRMM populations, confirming that the efficacy and safety of selinexor-based regimens are broadly reproducible across ethnicities and healthcare settings.

Collectively, real-world evidence from unselected populations validates that selinexor maintains stable efficacy and controllable safety across diverse routine clinical settings—including elderly, frail, renally impaired, and high-risk cytogenetic patients—providing more practical and generalizable clinical guidance than strictly screened trial data alone.

## 4 Spectrum of AEs and management strategies for selinexor

The unique mechanism of selinexor results in a characteristic, predictable, and manageable AE profile. These AEs primarily arise from on-target XPO1 inhibition in normal tissues and are consistent across trials (Table 2). A proactive, protocolized management strategy based on the “4P” framework (Prophylaxis, Prediction, Proactive monitoring, Prompt intervention) is essential for adherence and success (48, 57).

**Table 2:** Summary of AE incidence rates (%) in key clinical studies of selinexor for RRMM.

Study Name	Regimen	N	Thrombocytopenia (All/G3-4)	Neutropenia (All/G3-4)	Nausea (All/G3-4)	Fatigue (All/G3-4)
STORM (30)	Xd	122	73/60	21/21	72/7	73/23
BOSTON (31)	SVd	195	70/35	38/21	35/4	33/8
GEM-SELIBORDARA (44)	S-DVd	57	70/46	39/30	35/9	44/14
MARCH (58)	Xd	60	75/38	28/12	70/5	68/15

All/G3-4 = incidence of all-grade adverse events/incidence of grade 3–4 serious adverse events. All values are percentages. For the GEM-SELIBORDARA regimen, data on thrombocytopenia, neutropenia, nausea/vomiting and fatigue were extracted from the pooled safety data covering all 57 patients in the original publication (44).

### 4.1 Hematologic AEs

Hematologic toxicity is the most common AE requiring close monitoring during selinexor treatment and is a primary reason for dose adjustments. The integrated safety profile from 437 patients enrolled in clinical trials demonstrates that selinexor-associated hematologic AEs are predictable, manageable, and typically non-cumulative with appropriate dose modifications and supportive care (59).

Thrombocytopenia, the most frequent grade 3–4 hematologic toxicity (60% incidence in STORM) (30), is thought to result from XPO1 inhibition in megakaryocytes, where nuclear export of key transcriptional regulators is required for proper platelet production. Importantly, this effect is generally non-cumulative and reversible with dose interruption, distinguishing it from the myelosuppression caused by alkylating agents or prolonged proteasome inhibitor exposure (59). The predictable and reversible nature of this toxicity underpins the graded management approach described below. Platelet counts should be monitored at baseline and throughout treatment, with more frequent monitoring during the first 3 months of treatment. Consensus-based recommendations emphasize that proactive monitoring and early intervention are essential to optimize outcomes (60). The graded management strategy is summarized in Table 3.

**Table 3:** Graded management of thrombocytopenia.

Grade	Platelet Count	Clinical Presentation	Action (60)
Grade 1	75–99 × 10⁹/L	Asymptomatic	Maintain selinexor dose; continue weekly CBC monitoring
Grade 2	50–74 × 10⁹/L	Without bleeding	Maintain dose;continue weekly CBC monitoring; implement bleeding precaution education
Grade 3	25–49 × 10⁹/L	Without bleeding	Interrupt selinexor until recovery to ≤grade 2 (platelets ≥50 × 10⁹/L); upon recovery, restart at one dose level lower (e.g., 100 mg QW → 80 mg QW; 80 mg QW → 60 mg QW); consider thrombopoietin-receptor agonist (TPO-RA) (romiplostim 5–10 mcg/kg weekly or eltrombopag 50–150 mg daily) for persistent or recurrent cases
Grade 3	25–49 × 10⁹/L	With bleeding	Interrupt selinexor; consider platelet growth factors and transfuse per institutional guidelines; monitor until bleeding has stopped and platelet count returns to ≥50 × 10⁹/L; restart at 1 dose level lower
Grade 4	<25 × 10⁹/L	With or without bleeding	Immediately interrupt selinexor; administer platelet transfusion as clinically indicated; consider TPO-RA (romiplostim 1–10 mcg/kg SC weekly, or eltrombopag 50–150 mg daily); restart at reduced dose (typically 40 mg QW) upon recovery to ≥50 × 10⁹/L

Dose can be re-escalated when platelets have stabilized above 50 × 10⁹/L or baseline at physician discretion.

Neutropenia is also a common grade 3–4 AE (21%–33% incidence in BOSTON) (31). Regular monitoring is required. For grade 3 with fever or grade 4 neutropenia, treatment should be interrupted, and granulocyte colony-stimulating factor(G-CSF)(filgrastim 5 mcg/kg SC/IV daily, or pegfilgrastim 6 mg SC weekly) support administered, with treatment restarted at a reduced dose upon recovery.

Anemia occurs at a high overall incidence of approximately 80%, while the incidence of severe grade 3–4 anemia is relatively low and clinically manageable, ranging from 22% to 40% across major clinical trials (30, 31). Management should involve erythropoiesis-stimulating agents or transfusions based on clinical symptoms and hemoglobin levels.

### 4.2 Non-hematologic AE management

Non-hematologic AEs, although generally lower in severity, have a high incidence and significantly impact patient quality of life and treatment adherence. The integrated safety analysis confirmed that gastrointestinal and constitutional symptoms are the most frequently reported non-hematologic AEs, but they are rarely treatment-limiting when managed proactively (59).

Nausea/Vomiting is the most common non-hematologic AE (all-grade incidence ∼70%–80%, grade 3–4 < 10%) (30, 31). The high incidence of nausea and vomiting with selinexor is attributable to XPO1 expression in the gastrointestinal tract and the chemoreceptor trigger zone, where inhibition of nuclear export may affect neurotransmitter regulation. Unlike the myelosuppression seen with conventional chemotherapy, selinexor-associated gastrointestinal symptoms typically do not worsen with cumulative dosing; indeed, many patients experience symptom improvement after the first 8 weeks of therapy (48). This temporal pattern supports the use of intensive prophylaxis early in treatment with planned re-evaluation rather than indefinite continuation of antiemetic therapy. Prophylactic antiemetic administration is the cornerstone of management. The stepwise management approach (60) consists of upfront prophylaxis, lifestyle modification, and grade-based intervention, as described below.

Step 1 – Prophylaxis (before each selinexor dose): (i) All patients should receive a 5-HT3 receptor antagonist (e.g., ondansetron 8 mg PO, 30–60 min prior to the first dose of selinexor, with a subsequent 8 mg dose 8 h after the first dose; then 8 mg twice daily, covering 24 h after the last selinexor dose); (ii) For patients at high risk of nausea, add olanzapine 2.5–5.0 mg PO once daily in the evening or an NK1 receptor antagonist (e.g., aprepitant 125 mg day 1, then 80 mg days 2–3; or rolapitant per label); (iii) Some experts recommend starting with two antiemetic prophylactic medications—a 5-HT3 blocker plus either olanzapine or aprepitant—for optimal prevention; (iv) Continued use of prophylactic antiemetics should be evaluated after the first 8 weeks, as the incidence of nausea typically decreases over time.
Step 2 – Dietary and lifestyle adjustments: (i) Recommend small, frequent meals; avoid spicy, fatty, or strong-smelling foods; (ii) Consider evening dosing to allow patients to sleep through the peak nausea period; (iii)Maintain adequate hydration (at least 2 liters of electrolyte-containing fluids daily).
Step 3 – Management by grade is summarized in Table 4.

**Table 4:** Graded management of nausea/vomiting.

Severity	Presentation	Action (60)
Grade 1–2 nausea OR Grade 1–2 vomiting (≤5 episodes/day)	Mild to moderate	Maintain selinexor dose; add a second antiemetic from a different class (e.g., metoclopramide, prochlorperazine, or additional olanzapine)
Grade 3 nausea OR Grade ≥3 vomiting (>5 episodes/day)	Severe	Interrupt selinexor; monitor until nausea/vomiting resolves to ≤grade 2 or baseline; restart at one dose level lower

Fatigue/Anorexia has a high incidence (∼50%–70%) and severely affects quality of life. Nutritional support should be strengthened, encouraging small, frequent meals, and consulting a nutritionist when necessary, consider megestrol acetate 400 mg daily or low-dose olanzapine in the evening. For grade 2 weight loss (10% to <20%) or anorexia associated with significant weight loss or malnutrition, interrupt selinexor and institute supportive care; monitor until weight returns to ≥ 90% of baseline; restart at one dose level lower.

Hyponatremia is a notable metabolic AE (all-grade ∼40%, grade 3–4 ∼20%–22%) (30). Electrolytes should be monitored regularly during treatment. Asymptomatic hyponatremia(serum sodium 130–134 mmol/L) can be managed with oral sodium supplementation or salt tablets; dietary modification. For symptomatic or severe hyponatremia(<130 mmol/L or with neurologic symptoms), IV saline as needed; interrupt treatment if severe; monitor until sodium returns to ≥130 mmol/L; restart at one dose level lower.

A standardized institutional protocol based on the “4P” framework includes: (i) mandatory 5-HT3 antagonist before each dose for nausea prevention; (ii) weekly complete blood count for the first two cycles; (iii) baseline and weekly serum electrolytes; (iv) nutritional counseling for anorexia/fatigue; (v) pre-planned dose reduction algorithm for grade ≥3 toxicities. Implementation of such protocols has been shown to reduce grade 3–4 AE rates and treatment discontinuation (48, 57).

Although the 4P proactive management framework significantly improves the safety profile of selinexor, it still has obvious practical limitations in real-world clinical practice. The standardized dose-reduction algorithm is derived largely from clinical-trial datasets and lacks validated individualized adjustment indicators for special populations, such as dialysis-dependent patients and patients with concurrent autoimmune disorders. Moreover, current antiemetic and hematological supportive strategies predominantly address acute adverse events, whereas chronic low-grade fatigue and persistent metabolic disturbances occurring under long-term selinexor exposure remain incompletely resolved; these chronic toxicities constitute key barriers to sustained treatment adherence during maintenance-phase therapy (48, 57).

## 5 Summary and outlook

As the world's first oral SINE, selinexor has successfully provided a novel paradigm for RRMM treatment by innovatively targeting XPO1. Pivotal clinical studies confirm its efficacy, inducing deep responses and prolonging survival even in heavily pretreated, triple-class refractory patients. Critically, through a systematic, proactive management strategy emphasizing prophylaxis and individualized dose adjustment (e.g., QW regimens), its characteristic but predictable AEs have become controllable, transforming its tolerability profile in real-world practice.

**Limitations of this review:** First, the included clinical studies (e.g., STORM, BOSTON) are predominantly open-label and lack blinding, which may introduce bias in AE reporting. Second, sample sizes for certain subgroups (e.g., penta-refractory, extramedullary disease, GEM-SELIBORDARA quadruple-regimen cohort) are relatively small, limiting the generalizability of findings. Third, direct comparative data between selinexor-based regimens and other novel immunotherapies (e.g., CAR-T, bispecific antibodies) are currently unavailable, and cross-trial comparisons are confounded by differences in patient populations. Fourth, long-term safety data beyond 2–3 years are still emerging. **Strengths:** This review provides a comprehensive, up-to-date synthesis of selinexor's mechanism, clinical efficacy across diverse scenarios, and practical AE management strategies, with emphasis on real-world evidence and dose optimization—addressing a critical gap in clinical guidance for practicing hematologists.

Looking forward, the role of selinexor is poised to expand across multiple therapeutic dimensions. First, its application will be refined through biomarker-driven strategies (e.g., hnRNPU, p-STAT3, XPO1 mutation status) to personalize therapy, enabling more precise patient selection and improved risk-benefit profiles (28, 37, 40). Second, the treatment frontier is advancing into earlier lines, including frontline induction and maintenance therapy. Multiple ongoing phase 2/3 trials are evaluating all-oral selinexor-based regimens in these settings. The fully oral administration avoids infusion-related reactions and hospitalization, making it particularly suitable for long-term disease management. Preliminary data suggest that low-dose intermittent maintenance can suppress minimal residual disease, reduce recurrence in high-risk subgroups, and prolong PFS. The XVRd regimen has shown promising deep response rates (80% ≥VGPR) in newly diagnosed high-risk patients, warranting Phase 3 validation. Other all-oral combinations (e.g., with ixazomib and lenalidomide) are also under investigation, offering the potential for entirely outpatient-based, chemotherapy-free treatment. Third, its synergistic potential with emerging immunotherapies is a key horizon. Preclinical and early clinical evidence indicate that selinexor may enhance CAR-T efficacy by upregulating BCMA expression on myeloma cells and modulating the tumor microenvironment, potentially serving as an effective bridging regimen without compromising subsequent CAR-T outcomes (34, 42). Similarly, combining selinexor with bispecific antibodies (e.g., teclistamab, elranatamab) is theoretically attractive, as XPO1 inhibition may reduce T-cell exhaustion and improve immune synapse formation, though this strategy requires dedicated clinical validation (33, 34).

Compared with existing systematic reviews, this review further proposes two core research priorities for selinexor in the future. First, prospective large-sample cohort studies are urgently needed to verify the long-term efficacy and safety of weekly low-dose regimens in maintenance therapy, which is the key to promoting its long-term chronic management in MM. Second, the combination of selinexor with bispecific antibodies requires further mechanistic exploration and clinical validation, to clarify the optimal administration sequence and dose-matching strategy, and maximize the immune microenvironment regulatory advantage of XPO1 inhibition. Furthermore, cost-effectiveness evaluation of all-oral selinexor-based regimens is essential to improve drug accessibility in grassroots medical institutions.

However, several real-world challenges must be acknowledged alongside these promising developments. First, despite its broad application prospect, the widespread clinical popularization of selinexor remains constrained by high treatment costs, which limit drug accessibility—particularly in primary medical institutions and among low-income patient groups. While selinexor has received regulatory approval in the United States, Europe, and China, reimbursement policies vary considerably across countries and health systems, creating significant disparities in patient access. Second, chronic low-grade AEs—including persistent fatigue, mild gastrointestinal discomfort, and anorexia—can affect long-term medication adherence during maintenance therapy, even though these events are generally low-grade and manageable with supportive care. Strategies to support long-term compliance—including patient education, mobile health applications, and telemedicine follow-up—will be essential to optimize outcomes. Third, long-term follow-up data (≥3 years) regarding prolonged safety and real-world maintenance efficacy remain insufficient. The optimal duration of selinexor therapy remains undefined; ongoing studies are evaluating fixed-duration versus continuous treatment approaches to balance efficacy with long-term tolerability and cost-effectiveness. Future large-sample, long-term cohort studies are urgently needed to verify the sustained benefit and safety profile of selinexor in maintenance settings.

Ultimately, selinexor is evolving from an important late-line salvage drug into a foundational therapy, contributing significantly to the goal of long-term chronic disease management for multiple myeloma. However, realizing this potential will require continued efforts in biomarker discovery, combination optimization, and health-economic evaluation to ensure that this innovative therapy reaches all patients who may benefit from it.
